# Machine learning-based prediction models affecting the recovery of postoperative bowel function for patients undergoing colorectal surgeries

**DOI:** 10.1186/s12893-024-02437-9

**Published:** 2024-05-10

**Authors:** Shuguang Yang, Huiying Zhao, Youzhong An, Fuzheng Guo, Hua Zhang, Zhidong Gao, Yingjiang Ye

**Affiliations:** 1 Department of Critical Care Medicine, Peking University People’s Hospital, 11 Xizhimen South Street, Beijing, 100044 P.R. China; 2 Trauma Center, Peking University People’s Hospital, National Center for Trauma Medicine, Key Laboratory of Trauma and Neural Regeneration (Ministry of Education), Beijing, China; 3 Research Center of Clinical Epidemiology, Peking University Third Hospital, Xue Yuan Road, Haidian District, Beijing, 100191 P.R. China; 4 Laboratory of Surgical Oncology, Department of Gastrointestinal Surgery, Peking University People’s Hospital, 11 Xizhimen South Street, Beijing, 100044 P.R. China

**Keywords:** Machine learning, Time of first postoperative flatus, Time of first postoperative defecation, Colorectal surgeries, Prediction models

## Abstract

**Purpose:**

The debate surrounding factors influencing postoperative flatus and defecation in patients undergoing colorectal resection prompted this study. Our objective was to identify independent risk factors and develop prediction models for postoperative bowel function in patients undergoing colorectal surgeries.

**Methods:**

A retrospective analysis of medical records was conducted for patients who undergoing colorectal surgeries at Peking University People’s Hospital from January 2015 to October 2021. Machine learning algorithms were employed to identify risk factors and construct prediction models for the time of the first postoperative flatus and defecation. The prediction models were evaluated using sensitivity, specificity, the Youden index, and the area under the receiver operating characteristic curve (AUC) through logistic regression, random forest, Naïve Bayes, and extreme gradient boosting algorithms.

**Results:**

The study included 1358 patients for postoperative flatus timing analysis and 1430 patients for postoperative defecation timing analysis between January 2015 and December 2020 as part of the training phase. Additionally, a validation set comprised 200 patients who undergoing colorectal surgeries from January to October 2021. The logistic regression prediction model exhibited the highest AUC (0.78) for predicting the timing of the first postoperative flatus. Identified independent risk factors influencing the time of first postoperative flatus were Age (*p* < 0.01), oral laxatives for bowel preparation (*p* = 0.01), probiotics (*p* = 0.02), oral antibiotics for bowel preparation (*p* = 0.02), duration of operation (*p* = 0.02), postoperative fortified antibiotics (*p* = 0.02), and time of first postoperative feeding (*p* < 0.01). Furthermore, logistic regression achieved an AUC of 0.72 for predicting the time of first postoperative defecation, with age (*p* < 0.01), oral antibiotics for bowel preparation (*p* = 0.01), probiotics (*p* = 0.01), and time of first postoperative feeding (*p* < 0.01) identified as independent risk factors.

**Conclusions:**

The study suggests that he use of probiotics and early recovery of diet may enhance the recovery of bowel function in patients undergoing colorectal surgeries. Among the various analytical methods used, logistic regression emerged as the most effective approach for predicting the timing of the first postoperative flatus and defecation in this patient population.

**Supplementary Information:**

The online version contains supplementary material available at 10.1186/s12893-024-02437-9.

## Background

The recovery of bowel function after colorectal surgery has been extensively researched. Poor recovery of bowel function can lead to prolonged hospital stays, increased complications rates, higher hospitalization costs, and mortality [1, 2]. While stool form scales offer a straightforward approach to evaluating intestinal transit rate, they are not commonly utilized in clinical setting or research endeavors [3]. Symptoms like nausea and/or vomiting, fecal urgency, and bowel movement are considered indicative signals of postoperative bowel function restoration [4]. Time of first bowel motion, time of first postoperative flatus and defecation are employed to gauge postoperative bowel function for patients undergoing colorectal surgeries [5–8].

Numerous risk factors influence the recuperation of postoperative bowel function. Previous studies about recovery of bowel function following colorectal surgeries suggested that laparoscopic surgery may enhance postoperative bowel function recovery compared to traditional laparotomy [9]. Mechanical bowel preparation had shown benefits in some studies, yet recent research indicated that it may not consistently improve patients’ recovery and could lead to patients’ discomfort [10]. The outcomes concerning postoperative bowel function recovery remain contentious. Limited studies have delved into multivariate analysis of the time of the first postoperative flatus and defecation for patients undergoing colorectal surgeries. Furthermore, no study had developed a prediction model for postoperative bowel function recovery in this patient population.

This study aimed to establish prediction models using machine learning algorithms to assess the risks and identify independent risk factors associated with the time of first postoperative flatus and defecation for patients undergoing colorectal surgeries.

## Methods

### Participants

This study was approved by the Ethics committee of Peking University People’s Hospital (2022PHB053-001, Beijing, China). A retrospective study was conducted to develop and internally validate the time of the first postoperative flatus and defecation. Adult patients undergoing colorectal surgeries at Peking University People’s Hospital from January 2015 to October 2021 were enrolled. Exclusion criteria were patients who met one of the following characteristics:

- Patients who had a history of surgical reconstruction of the digestive tract.
- Patients who had undergone enterostomies, such as jejunostomy, total proctocolectomy with ileostomy, or colostomy.
- Patients who were younger than 18 years old.
- Data on the time to postoperative flatus or/and defecation were incomplete.

Previous studies [11, 12] found that the mean time of the first postoperative flatus was 4 days and the time of the first postoperative defecation was 5 days for patients undergoing colorectal surgeries. Therefore, we defined patients in two groups between the time of the first postoperative flatus within 4 days and more than 5 days. We defined patients in two groups between the time of the first postoperative defecation within 4 days and more than 5 days as well.

### Data collection

General data of patients were carefully recorded, including age, gender, body mass index (BMI), history of alcohol, and history of smocking. Underlying disease (hypertension, coronary heart disease [CHD], arrhythmia, cerebral infraction, encephalorrhagia, hypothyroidism, diabetes, chronic obstructive pulmonary disease [COPD], renal inadequacy, hyperlipidemia, hepatic inadequacy, blood disease) diagnosed before admission were entered into excel of case report form (CRF). Data of preoperative chemotherapy, preoperative anemia, preoperative ileus, the American Society of Anesthesiologists (ASA) classification, bowel preparation before surgery (soapsuds enema, oral laxatives, glycerin enema, and oral antibiotics) were adopted in CRF. Data of surgery such as surgical site (right hemicolectomy, transverse colectomy, left hemicolectomy, sigmoid colectomy, and proctectomy), surgical approach (laparotomy or laparoscopic surgery), excision method (local resection or extended radical resection), duration of operation, antibiotics correlation (preoperative, fortified before surgery, fortified after surgery, duration of antibiotics) were entered into CRF. Postoperative data including time of the first postoperative feeding (day), probiotics correlation; postoperative albumin level, postoperative analgesia (no analgesia, opioids, opioids combine non-steroidal anti-inflammatory drugs [NSAIDs], and take NSAIDs alone), duration of analgesia, time to the extraction of a gastric tube and the drainage tube were entered recorded. All data were obtained by medical record such as papery medical records library or electronic medical record system.

### Statistical analysis

#### Univariate analysis

Univariate analysis was performed using SPSS 26.0 software (IBM, Armonk, NY, USA) to identify the relative risk factors affecting the time of first postoperative flatus and defecation by. Quantitative data with normal distribution were expressed as mean ± standard deviations (SD) or medians and interquartile ranges and compared using a one-way analysis of variance. Frequencies and percentages were used for categorical variables. An independent sample* t-*test was performed according to the homogeneity of variance for continuous variables. The frequency and composition ratio were used for the statistical description of classification data, and the χ^2^ test or Fisher’s exact test was used for comparison between groups. A *p*-value < 0.05 was considered to indicate significance.

#### Model development

Prediction models for the time of the first postoperative flatus and defecation were developed using four machine learning algorithms: logistic regression (LR), random forest (RF), Naïve Bayes (NB), and extreme gradient boosting (XGB). Data from patients undergoing colorectal surgeries from January 2015 to December 2020 were used as training sets, while data from January to October 2021 served as validation sets. We calculated the number of true-positive (TP), true-negative (TN), false-positive (FP), and false-negative (FN) results. Performance and discrimination of the prediction models were evaluated by the area under the receiver operating characteristic curve (AUC), sensitivity, specificity, positive predictive value (TP/[TP + FP]), negative predictive value (TP/[TP + FN]), and Youden index ([sensitivity + specificity]-1). The AUC value greater than 0.6 indicated good predictive value, with closer value to 1 indicating better model performance. Nomograms based on the results of logistic regression were planned if logistic regression outperformed the other three methods. The prediction models were developed using the R software RMS package (version 4.0.3).

## Results

### Baseline characteristics and related risk factors

**Fig. 1:**
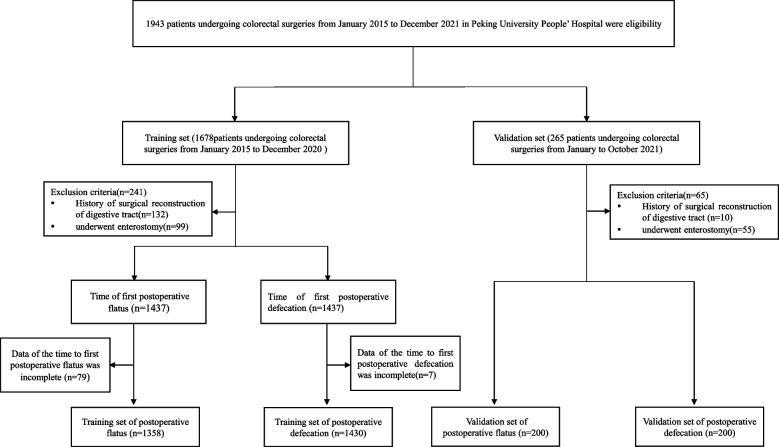
Study eligibility of patients who undergone colorectal surgeries

A total of 1438 patients undergoing colorectal surgeries from January 2015 to December 2020 were involved, including 856 patients for the time of first postoperative flatus within 4 days and 1052 patients for the time of first postoperative flatus within 5 days in the training set. 200 patients undergoing colorectal surgeries from January to October 2021 were involved in the validation set (Fig. 1). The mean time to postoperative flatus was 4.17 ± 1.45 days and the mean time to postoperative defecation was 4.77 ± 1.89 days.

#### Time of first postoperative flatus

Clinical characteristics of the patients at the time of the first postoperative flatus were shown in Table 1. Among univariate analysis, age, right colectomy, sigmoid colectomy, malignancy, hypothyroidism, preoperative anemia, preoperative ileus, ASA classification, soapsuds enema, oral laxatives, and oral antibiotic for bowel preparation, laparotomy, duration of operation, preoperative antibiotics, preoperative fortified antibiotics, time of postoperative feeding, probiotics, duration of analgesia, hypoproteinemia, time to the extraction of the gastric tube and drainage tube were associated with the time of first postoperative flatus for patients undergoing colorectal surgeries.

**Table 1:** Basic characteristics in the time of first postoperative flatus for patients undergoing colorectal surgeries

Variables N(%)	Group 1 (≤ 4 day)	Group 2(> 4 day)	*χ*^*2*^*/ t*	*P* value
	*n* = 856	*n* = 502		
Female	396(46.30)	246(49.00)	0.96	0.33
Age (years)	62.08 ± 13.00	66.14 ± 12.95	-4.58	< 0.01
BMI^a^	23.62 ± 3.54	23.53 ± 3.47	0.43	0.67
Right colectomy	266(31.10)	184(36.70)	4.45	0.04
Transverse colectomy	46(5.40)	21(4.20)	0.96	0.33
Left colectomy	81(9.50)	56(11.20)	1.00	0.32
Sigmoid colectomy	328(38.30)	156(31.10)	7.24	0.01
Proctectomy	196(22.90)	106(21.10)	0.58	0.45
Malignancy	808(94.40)	487(97.00)	4.91	0.03
Hypertension	545(63.70)	300(59.80)	2.06	0.15
CAD^b^	107(12.50)	76(15.10)	1.89	0.17
Arrhythmia	54(6.30)	38(7.60)	0.80	0.37
Cerebral infarction	60(7.00)	50(10.00)	3.70	0.05
Encepalorrhagia	6(0.700)	4(0.800)	0.40	0.84
Hypothyroidism	11(1.30)	17(3.40)	6.92	0.01
Diabetes mellitus	157(18.30)	93(18.50)	0.01	0.93
COPD^c^	37(4.30)	23(4.60)	0.05	0.82
Renal inadequacy	18(2.10)	16(3.20)	1.52	0.22
Hyperlipidemia	185(21.60)	95(18.90)	1.40	0.24
Hepatic inadequacy	15(1.80)	5(1.00)	1.25	0.26
Blood disease	10(1.20)	3(0.60)	1.09	0.30
History of alcohol	109(12.70)	75(14.90)	1.32	0.25
History of smocking	141(16.50)	90(17.90)	0.48	0.49
Preoperative chemotherapy	32(3.70)	23(4.60)	0.58	0.45
Preoperative anemia	356(41.60)	248(49.40)	7.82	0.01
Preoperative ileus	154(18.00)	116(23.10)	5.20	0.02
ASA I^d^	115(13.40)	39(7.80)	20.57	< 0.01*
ASA II	621(72.50)	355(70.70)		
ASA III	116(13.60)	102(20.30)		
ASA IV	4(0.50)	6(1.20)		
Soapsuds enema for bowel preparation	70(8.20)	65(12.90)	8.04	< 0.01
Oral laxatives for bowel preparation	805(94.00)	443(88.20)	14.28	< 0.01
Glycerin enema for bowel preparation	127(14.80)	89(17.70)	1.98	0.12
Oral antibiotic for bowel preparation	27(3.20)	32(6.40)	7.90	0.01
Laparotomy	399(46.60)	286(57.00)	13.59	< 0.01
Extended radical resection	773(90.30)	421(83.900)	12.36	< 0.01
Duration of operation (min)	203.07 ± 60.60	3215.71 ± 76.38	-3.17	< 0.01
Preoperative antibiotics	43(5.00)	44(8.80)	7.39	0.01
Preoperative fortified antibiotics	24(2.80)	26(5.20)	5.04	0.03
Postoperative fortified antibiotics	363(43.40)	234(46.60)	2.27	0.13
Duration of antibiotics (day)	7.02 ± 5.10	7.51 ± 4.31	-1.80	0.07
Time of postoperative feeding (day)	5.19 ± 2.10	6.55 ± 2.23	-10.19	< 0.01
Probiotics	258(27.60)	119(20.40)	6.53	0.01
Postoperative analgesia	806(94.2)	477(95.00)	0.45	0.50
None	50(5.80)	25(5.00)	2.11	0.55*
Opioids	749(87.50)	444(88.40)		
Opioids & NSAIDs^e^	46(5.40)	30(6.00)		
NSAIDs	11(1.30)	3(0.60)		
Duration of analgesia (day)	2.73 ± 1.02	2.91 ± 1.10	-3.10	< 0.01
Hypoproteinemia	688(80.40)	442(88.00)	13.34	< 0.01
Extraction of gastric tube (day)	1.69 ± 3.80	2.15 ± 2.43	-2.73	0.01
Extraction of drainage tube (day)	8.59 ± 5.98	9.68 ± 8.60	-2.72	0.01

^a^*BMI* body mass index, ^b^*CHD* coronary heart disease, ^c^*COPD* chronic obstructive pulmonary disease, ^d^*ASA* American Society of Anesthesiologists, ^e^*NSAIDs* non-steroidal anti-inflammatory drugs

^*^Comparison between two groups, *group and*group. The difference between groups was statistically significant (*P* < 0.01 or *P* < 0.05)

#### Time of first postoperative defecation

We investigated the variates by univariate analysis and found that 14 indicators including age, right hemicolectomy, proctectomy, encepalorrhagia, preoperative chemotherapy, ASA classification, glycerin enema for bowel preparation, oral antibiotics for bowel preparation, preoperative antibiotics, preoperative fortified antibiotics, time of first postoperative feeding, probiotics, hypoproteinemia and time to the extraction of the drainage tube were associated with that prolong the time of first postoperative defecation (Table 2).

**Table 2:** Regression coefficients of the time of first postoperative flatus model based on 7 independent variables

	OR (95%CI)	*P* value
Age	1.00(1.00–1.00)	< 0.01
Oral laxatives for bowel preparation		0.01
No	1.00	
Yes	0.60(0.40–0.90)	
Oral antibiotics for bowel preparation		0.02
No	1.00	
Yes	2.00(1.10–3.60)	
Probiotics		0.02
No	1.00	
Yes	0.02(0.75–0.95)	
Postoperative fortified antibiotics		0.02
No	1.00	
Yes	1.40(1.00–1.80)	
Duration of operation(min)	1.00(1.00–1.00)	0.02
Time of postoperative feeding (day)	1.30(1.30–1.40)	< 0.01

### Development of prediction models

#### Time of first postoperative flatus

**Fig. 2:**
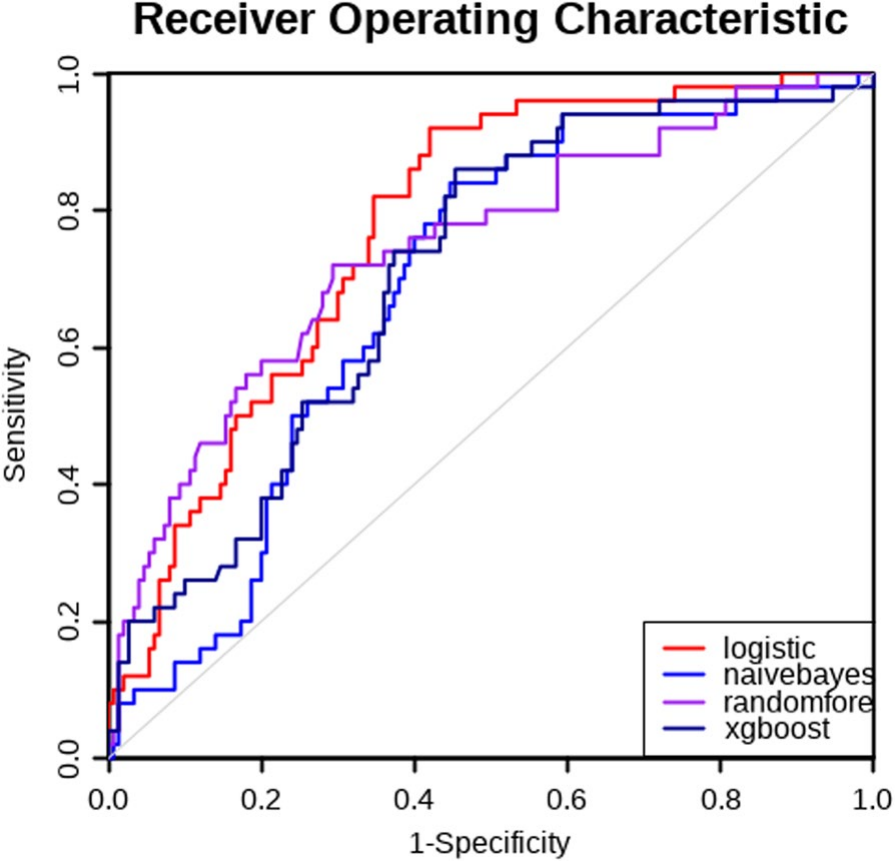
Receiver operating characteristic (ROC) curves of the prediction model for the time of first postoperative flatus conducted by machine learning algorithms

**Fig. 3:**
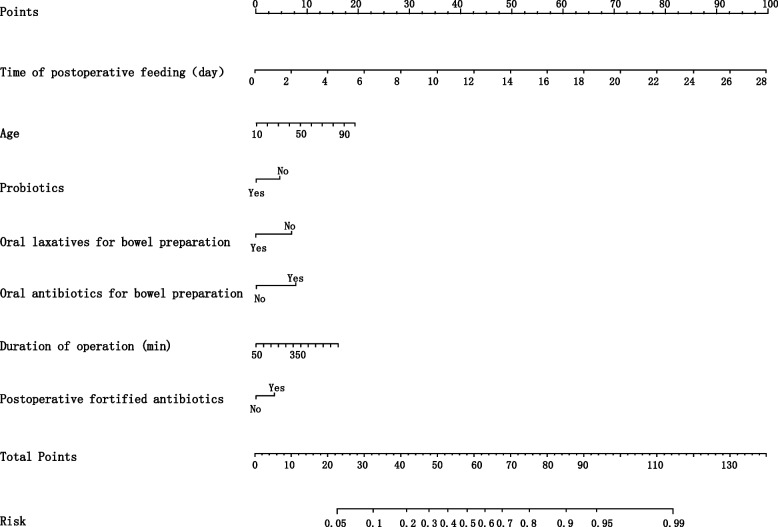
Nomogram for the time of first postoperative flatus. To estimate the probability of the time of postoperative flatus, mark patient value at each axis, draw a straight line perpendicular to the point axis, and calculate the points for all variables. Then mark the sum on the total point axis and the points met the risk axis

Four prediction models were conducted based on the aforementioned variables by machine learning algorithms. We used the data from January 2015 to December 2020 as a training set and the samples from January to October 2021 as a validation set. The area under the receiver operating characteristic (AUC) was 0.78(0.71–0.84) in the validation of logistic regression analysis, 0.74(0.66–0.83) in the validation of random forest (RF), 0.69(0.61–0.77) in the validation of Naïve Bayes (NB), and was 0.71(0.63–0.79) in the validation of extreme gradient boosting (XGB) for the prediction model of the time to postoperative flatus for patients undergoing colorectal surgeries (Fig. 2). Logistic regression was found to be the best-performing model for predicting the time of the first postoperative flatus comparing with the other three models as the AUC, sensitivity, specificity, positive predictive value (PPV), negative predictive value (NPV) and Youden index (sensitivity + specificity-1) were shown in Additional file 1. A nomogram was used to present the data of the time to the first postoperative flatus based on logistic regression for practical use (Fig. 3). A total number of points was calculated with age, probiotics, oral laxatives for bowel preparation, oral antibiotics for bowel preparation, duration of operation, and time of first postoperative feeding. The total score can be attached to the probability of the time to postoperative flatus (Tables 3 and 4).

**Table 3:** Basic characteristics in the time of first postoperative defecation for patients undergoing colorectal surgeries

Variables N(%)	Group 1(≤ 5 day)	Group 2 (> 5 day)	*χ*^*2*^*/ t*	*P* value
	*n* = 1052	*n* = 378		
Female	504(47.90)	177(46.80)	0.13	0.71
Age (years)	63.39 ± 13.16	66.69 ± 12.45	-4.25	< 0.01
BMI^a^	23.56 ± 3.49	23.66 ± 3.58	-0.49	0.62
Right colectomy	379(36.00)	103(27.20)	9.59	< 0.01
Transverse colectomy	58(5.50)	13(3.40)	2.54	0.11
Left colectomy	101(9.60)	40(10.60)	0.30	0.58
Sigmoid colectomy	374(35.60)	127(33.60)	0.47	0.50
Proctectomy	207(19.70)	114(35.50)	7.55	< 0.01
Malignant	1000(95.10)	365(96.60)	1.45	0.23
Hypertension	653(62.10)	231(61.10)	0.11	0.74
CAD^b^	131(12.50)	61(16.10)	3.25	0.07
Arrhythmia	66(6.30)	30(7.90)	1.23	0.27
Cerebral infarction	85(8.10)	35(9.30)	0.50	0.48
Encepalorrhagia	4(0.40)	6(1.60)	5.84	0.02
Hypothyroidism	19(1.80)	12(3.20)	2.46	0.12
Diabetes mellitus	204(18.40)	61(18.60)	0.00	0.95
COPD^c^	45(4.30)	20(5.30)	0.66	0.42
Renal inadequacy	24(2.30)	13(3.40)	1.48	0.22
Hyperlipidemia	220(20.90)	72(19.00)	0.60	0.44
Hepatic inadequacy	15(1.40)	5(1.30)	0.02	0.88
Blood disease	10(1.00)	3(0.80)	0.08	0.78
History of alcohol	144(13.70)	47(12.40)	0.38	0.54
History of smocking	181(17.20)	63(16.70)	0.06	0.81
Preoperative chemotherapy	36(3.40)	22(5.80)	4.11	0.04
Preoperative anemia	469(44.60)	166(43.90)	0.05	0.82
Preoperative ileus	199(18.90)	82(21.70)	1.36	0.24
ASA I^d^	133(12.60)	30(7.90)	10.34	0.02*
ASA II	759(72.10)	270(71.40)		
ASA III	152(14.40)	74(19.60)		
ASA IV	8(0.80)	4(1.10)		
Soapsuds enema for bowel preparation	106(10.10)	442(11.60)	0.73	0.40
Oral laxative bowel preparation	968(92.00)	338(89.40)	2.37	0.12
Glycerin enema for bowel preparation	150(14.30)	84(22.20)	12.89	< 0.01
Oral antibiotics for bowel preparation	35(3.30)	27(7.10)	9.76	< 0.01
Laparotomy	532(50.60)	200(52.90)	0.61	0.44
Extended radical resection	923(87.70)	329(87.00)	0.13	0.72
Operative time (min)	206.96 ± 63.13	211.02 ± 79.39	-0.90	0.37
Preoperative antibiotics	61(5.80)	36(9.50)	6.10	0.01
Preoperative fortified antibiotics	34(3.20)	23(6.10)	5.91	0.02
Postoperative fortified antibiotics	456(43.30)	155(41.00)	0.62	0.43
Duration of antibiotics (day)	7.10 ± 4.86	7.65 ± 4.68	-1.93	0.05
Time of postoperative feeding (day)	5.38 ± 1.92	6.59 ± 2.75	-7.08	< 0.01
Probiotics	305(29.00)	78(20.60)	9.91	< 0.01
Postoperative analgesia	996(94.70)	357(94.40)	0.03	0.86
None	56(5.32)	22(5.82)		
Opioids	930(88.40)	332(87.84)		
Opioid & NSAIDs^e^	53(5.04)	23(6.08)		
NSAIDs	13(1.24)	1(0.26)		
Duration of analgesia	2.77 ± 1.00	2.85 ± 1.19	-1.14	0.25
Hypoproteinemia	867(82.40)	378(87.00)	4.34	0.04
Extraction of gastric tube (day)	1.77 ± 3.53	2.16 ± 2.57	-1.94	0.05
Extraction of drainage tube (day)	8.73 ± 6.93	9.98 ± 7.12	-3.00	< 0.01

^a^*BMI* body mass index, ^b^*CHD* coronary heart disease, ^c^*COPD* chronic obstructive pulmonary disease, ^d^*ASA* American Society of Anesthesiologists, ^e^*NSAIDs* non-steroidal anti-inflammatory drugs

^*^Comparison between two groups*group and*group. The difference between groups was statistically significant (*P* < 0.01 or *P* < 0.05)

**Table 4:** Regression coefficients of the time of first postoperative defecation model based on 4 independent variables

	OR (95%CI)	*P* value
Age	1.00(1.00–1.00)	< 0.01
Oral antibiotics for bowel preparation		0.01
No	1.00	
Yes	2.10(1.20–3.70)	
Probiotics		0.01
No	1.00	
Yes	0.60(0.50–0.90)	
Time of postoperative feeding (day)	1.30(1.20–1.30)	< 0.01

#### Time of first postoperative defecation

**Fig. 4:**
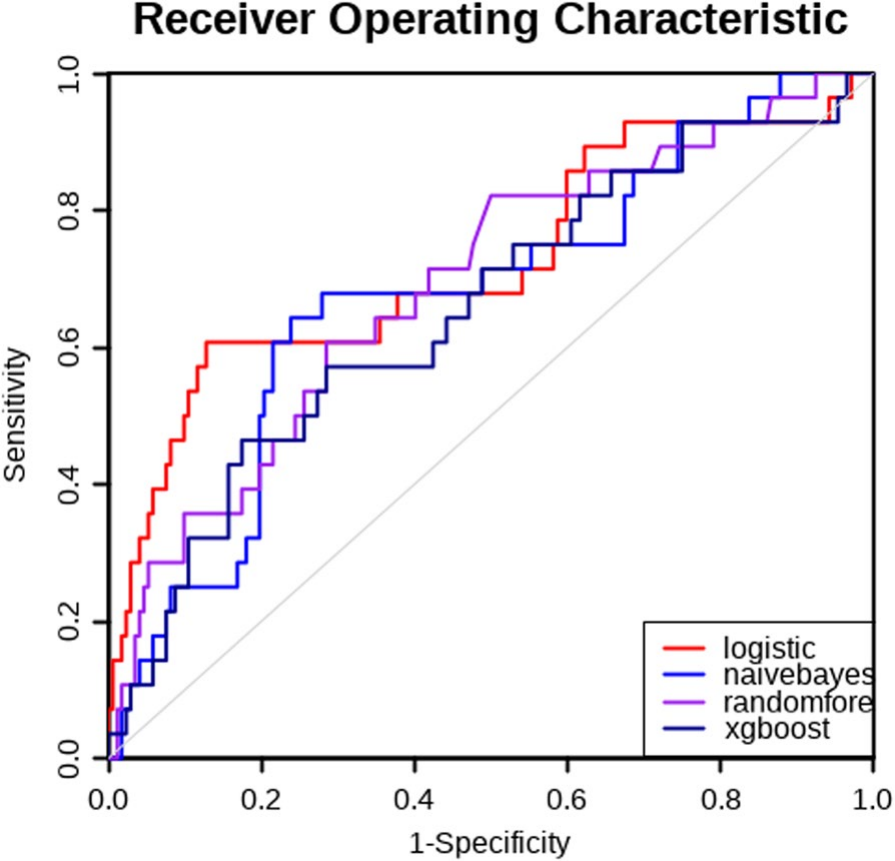
Receiver operating characteristic (ROC) curves for the prediction model of the time of first postoperative defecation conducted by machine learning algorithms

**Fig. 5:**
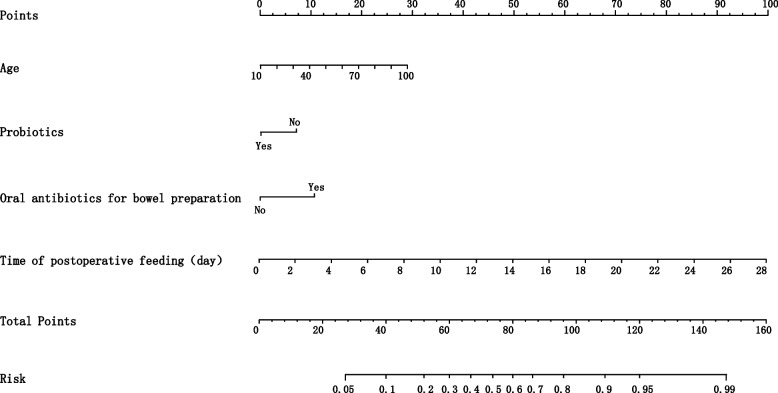
Nomogram for the time of first postoperative defecation. The value of variable was given a score on the point scale axis. To estimate the risk of the time of first postoperative defecation, a total score could be calculated by each axis and could be projected to the lower total point scale

In the validation set, AUCs for the LR, RF, NB, and XGB algorithms were 0.72(0.61–0.84), 0.69(0.58–0.80), 0.68(0.57–0.79), and 0.66 (0.54–0.77) (Fig. 4). The performance of AUC, sensitivity, specificity, PPV, NPV, and Youden index was summarized in Additional file 2. We selected the LR algorithm for the final model because the prediction model of the time to the first postoperative defecation performed well by LR. A nomogram for the time of the first postoperative defecation using by LR for patients undergoing colorectal surgeries was created based on the independent risk factors. The value of age, probiotics, oral antibiotics for bowel preparation, and time of postoperative feeding was given a score on the point scale axis in Fig. 5. A total score can be calculated by adding each score of these independent risk factors to estimate the probability of the time to the first postoperative defecation.

#### Postoperative complications

The incidence of postoperative complications was shown in Table 5. The symptom of abdominal distension (27.97%) contributed the highest rate of postoperative complications, while the incidence of diarrhea (27.70%) placed second to postoperative complications for patients undergoing colorectal surgeries.

**Table 5:** Postoperative complications of patients undergoing colorectal surgeries

Postoperative complications	n (%)
Vomiting	100(6.96)
Abdominal distension	402(27.97)
Diarrhea	398(27.70)
Wound infection	54(3.76)
Postoperative pneumonia	41(2.85)
Postoperative bowel obstruction	153(10.65)
Chlufitula	18(1.25)
Intestinal leakage	33(2.30)

## Discussion

In this study, we evaluated the ability of four machine learning algorithms to predict the time of postoperative flatus and defecation for patients undergoing colorectal surgeries. Our final prediction model achieved an AUC value of 0.78(0.71–0.84) for the time of postoperative flatus and 0.72(0.61–0.84) for the time of postoperative defecation according to the best performance of the logistic regression model compared with the other three models. The logistic regression model identified seven variables age, oral laxatives for bowel preparation, oral antibiotics for bowel preparation, probiotics, postoperative fortified antibiotics, duration of operation, and time of postoperative feeding for the time of postoperative flatus and four variables age, oral antibiotics for bowel preparation, probiotics, and time of postoperative feeding for the time of postoperative defecation.

The function of the bowel is to ingest and digest food and fluids, absorb nutrients, and eliminate any waste products, which is important to understand how surgery may alter not just its anatomy, but also its function [13]. Postoperative recovery is a dynamic process in that patients try to regain their independence, but postoperative bowel dysfunction is one of the most common complications among patients who have undergone major abdominal surgery [14]. Urinary and sexual dysfunction are the most common complications for patients undergoing rectal surgery [15]. Bowel dysfunction can manifest as constipation, anal incontinence, or diarrhea.is more likely to occur if there is a large bowel resection such as a colectomy or if most of the rectum is resected [16]. Regaining normal bowel functions after surgery is considered important for patients. Bowel motion, the time of first postoperative flatus, and the time of first postoperative defecation are usually used to assess bowel function during early postoperative recovery [17]. In this study, the time of postoperative flatus and defecation were selected to assess postoperative bowel function.

We found that mechanical bowel preparation with antibiotics and age were strong predictors for the risk of postoperative flatus and defecation. The mean age of the patients was 64.03 years in the study about the time of postoperative flatus and the mean age of patients with the time of postoperative flatus more than 5 days was 67.05 years in this study. Mechanical bowel preparations such as soap enema and oral laxatives can reduce fecal bulk which may decrease bacterial colonization, thereby reducing the risk of postoperative complications such as anastomotic leakage and surgical site infection [18]. The studies told us that mechanical bowel preparation combined with oral antibiotic bowel preparation can reduce the incidence of surgical site infection, anastomotic leakage, and other morbidity compared with mechanical bowel preparation for patients undergoing elective colorectal surgery [19–23]. Therefore, it was recommended that mechanical bowel preparation combined with oral antibiotic preparation for patients undergoing elective colorectal surgery in 2009 US guidelines [24]. In recent years, many studies found that the potential advantages of mechanical bowel preparation combined with an oral antibiotic, such as nausea and dehydration were considered not worthwhile [25] and did not add significant value to reducing the incidence of infectious complications [26].

Probiotics and the time of postoperative feeding were predictors of reducing the time of postoperative flatus and defecation in this study. Many researchers have focused on probiotics because they found that gastrointestinal microflora plays an important role in maintaining human health [27]. Probiotics help to improve the intestinal microecology balance and stimulate immunity, which may inhibit colon cancer and decrease the incidence of postoperative complications including surgical site infection, urinary tract infection, and septicemia [28–30]. Early enteral nutrition is recommended for patients after gastrointestinal surgery [31–33]. A study about postoperative feeding for patients undergoing colorectal surgeries found that there was no difference between patients who accepted early postoperative feeding and traditional postoperative feeding [34]. Early resume to postoperative feeding helps improve clinical outcomes such as promoting bowel motility, shortening the time of postoperative defecation, and reducing intestinal mucosal hypermetabolism [35, 36].

Previous studies comparing traditional open and laparoscopic surgery for rectal cancer had found that mean time about the time of postoperative flatus was 96.5 h vs 123 h [9]. Recent research showed that there was no difference of time to recovery of postoperative bowel function among different site of colon [37]. Another study suggested that robotic reduced-port surgery for left-sided colorectal cancer was safe and no additional benefit compared with laparoscopic surgery [38]. In our study, there was no difference between laparotomy and laparoscopic surgery of colorectum for patients undergoing colorectal surgery. The Enhanced Recovery after Surgery (ERAS) protocol was well developed especially for patients undergoing the surgery treatment in laparoscopic colorectal tumor resection since the ERAS Study formed in Europe in 2001 [39]. The data varied widely because there were different wards in the department of gastrointestinal surgery. More and more surgeons are following the principle of ERAS protocol for perioperative management. However, there were also someone choose traditional methods for perioperative management in our department.

In recent years, artificial intelligence has mostly narrowed down to machine learning methods. Current machine learning methods include neural networks, support vector machines, or random forests that have been used to develop prediction models and identify risk factors in recent years [40, 41], but statistical models have limitations in processing numerous unrefined variables. In this study, LR showed the best performance among the other three prediction models because the assessment indicator of postoperative bowel function was limited. We believe that machine learning algorithms will be actively used as tools for predicting complex outcomes and have greater potential.

There were several limitations in this study. Firstly, the variables of the models are clinically relevant, but causality cannot be confirmed due to the nature of retrospective data. Secondly, due to retrospective design, possible collection, entry bias, and residual confounding may occur, and we did not collect the medical history of constipation. Furthermore, the risk of the time of postoperative flatus and defecation is complicated. Thirdly, our study is a single-center study due to the lack of data from other surgical centers. We validated our model by different time at the same independent dataset, which is considered to be a kind of controversial external validation. Despite these limitations, ours is the first study to identify independent risk factors for the time of postoperative flatus and defecation in colorectal surgeries using a machine learning algorithm.

## Conclusion

By means of machine learning techniques, we selected independent risk factors, as well as evaluated prediction models for the first postoperative flatus and defecation time on adult patients undergoing colorectal surgeries. In addition, probiotics and early recovery of postoperative feeding may improve postoperative bowel function, while oral antibiotics for bowel preparation may affect postoperative bowel function for those patients.

### Supplementary Information

Supplementary Material 1.

## Data Availability

The data are available from the corresponding author on reasonable request. But the datasets are not publicly available due to privacy or ethical restrictions.

## Abbreviations

BMI: Body mass index
CHD: Coronary heart disease
COPD: Chronic obstructive pulmonary disease
ASA: American Society of Anesthesiologists
NSAIDs: Non-steroidal anti-inflammatory drugs
CRF: Case report form
OR: Odds ratios
CI: Confidence intervals
LR: Logistic regression
RF: Random forest
NB: Naïve Bayes
XGB: Extreme gradient boosting
TP: True-positive
TN: True-negative
FP: False-positive
FN: False-negative
ROC: Receiver operating characteristic
AUC: Area under the receiver operating characteristic curve
PPV: Positive predictive value
NPV: Negative predictive value
