# Supplementary material for: Machine learning-based prediction models affecting the recovery of postoperative bowel function for patients undergoing colorectal surgeries
Source: BMC Surg. 2024 May 10;24:143. doi: 10.1186/s12893-024-02437-9 (PMC11088159; doi:10.1186/s12893-024-02437-9)

**Additional file 1 Performance of four prediction model for the time of first postoperative flatus. A: area under the receiver operating characteristic curve (AUC) of prediction models, B: sensitivity, specificity, PPV, NPV, and Youden Index of prediction models. PPV: positive predictive value, NPV: negative predictive value**


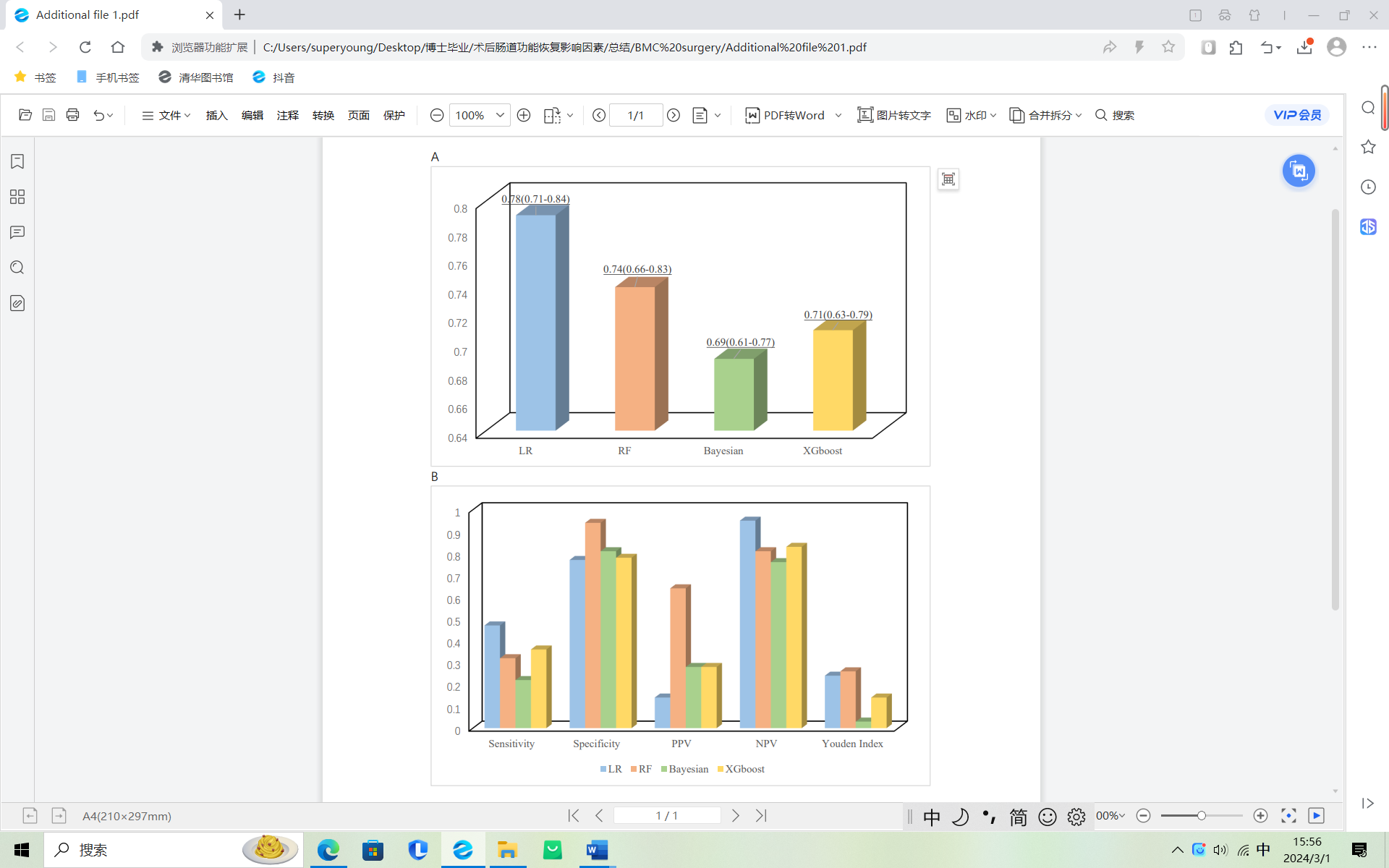


**Additional file 2 Performance of four prediction model for the time of first postoperative defecation. A: area under the receiver operating characteristic curve (AUC) of four prediction models, B: sensitivity, specificity, PPV, NPV, and Youden Index of four prediction models. PPV: positive predictive value, NPV: negative predictive value**


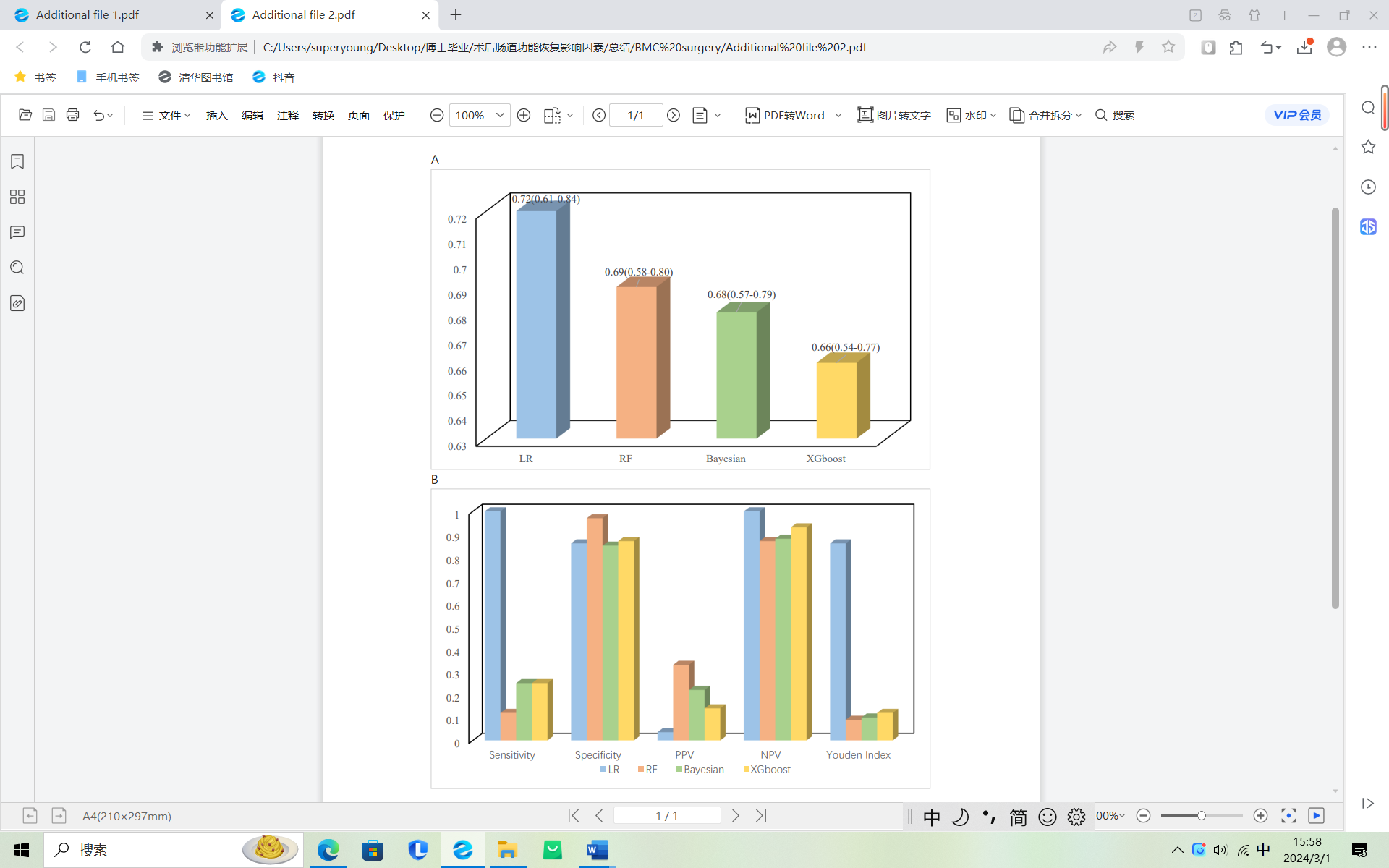

Supplement: Supplementary file 1 — Supplementary Material 1. [file 12893_2024_2437_MOESM1_ESM.docx]
